# From plaques to pigment: Eruptive lentiginosis in resolving psoriatic plaques after biologic therapy

**DOI:** 10.1016/j.jdcr.2025.09.045

**Published:** 2025-10-25

**Authors:** Sarah Choe, Aneri Bhargav Patel, Celine Phong, Katerina Yale, Nicole M. Golbari, Christopher B. Zachary, Michelle S. Min

**Affiliations:** a Department of Dermatology, University of California Irvine School of Medicine, Irvine, California; b California University of Science and Medicine, School of Medicine, Colton, California; c University of California, Davis, School of Medicine, Sacramento, California; d Department of Dermatology, New York University Langone Health, New York, New York

**Keywords:** biologic therapy, hyperpigmentation, lentigines, psoriasis

## Introduction

Eruptive lentiginosis arising in areas of resolving psoriatic plaques (ELRP) is a rare phenomenon, first described in 1994 following treatment with ultraviolet A phototherapy.^1^^,^^2^ Since this initial report, subsequent cases have emerged in association with various therapeutic modalities for psoriasis, including topical corticosteroids, calcipotriol, coal tar solution, oral methotrexate, and apremilast.^3^ More recently, biologic agents have been implicated in an increasing number of ELRP cases.^2^^,^^4^, ^5^, ^6^, ^7^, ^8^, ^9^ Herein, we present 6 cases of ELRP following treatment with either an interleukin (IL) −17A or IL-23 inhibitor. To further understand this phenomenon, a comprehensive literature review was completed to identify potential risk factors for the development of ELRP subsequent to initiation of psoriasis biologic therapy.

## Case series

### Case 1

**Fig 1:**
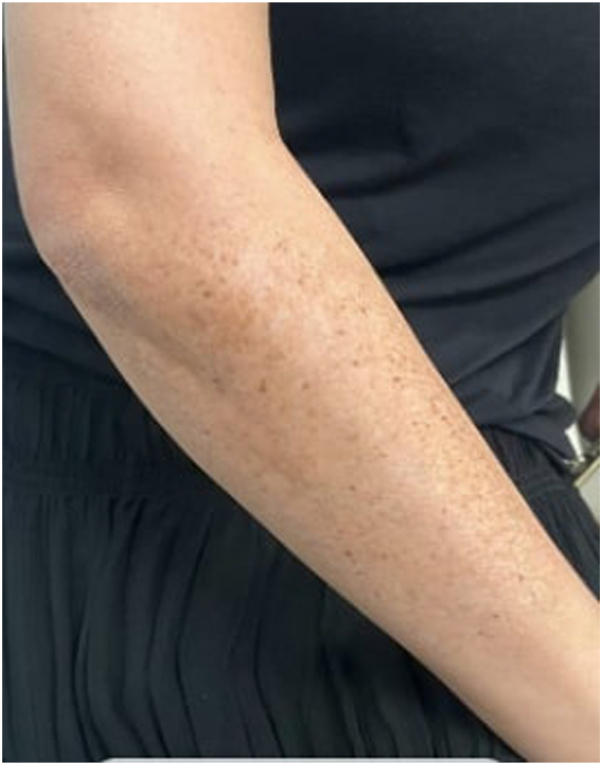
Clinical images of Patient 1, demonstrating speckled lentigines at the site of previous psoriatic plaques on the right extensor forearm.

A 51-year-old woman with Fitzpatrick skin type (FST) III and a history of anxiety, hyperlipidemia (HLD), hypertension (HTN), obesity, and psoriatic arthritis (PsA) presented with a 10-year history of severe psoriasis on her scalp, neck, back, abdomen, upper extremities, chest, and thighs. She was on secukinumab (150 mg every 4 weeks), started 1 year prior. Previous treatments included topicals, acitretin, narrowband ultraviolet B (NB-UVB) phototherapy, and adalimumab, all of which provided minimal improvement of psoriatic plaques. Upon initiation of secukinumab, the patient’s psoriasis. On physical examination (PE), faint hyperpigmented patches were observed on bilateral forearms and elbows, corresponding to sites of previous psoriatic plaques. Within the patches, multiple 2-4 mm light brown macules were observed (Fig 1).

### Case 2

**Fig 2:**
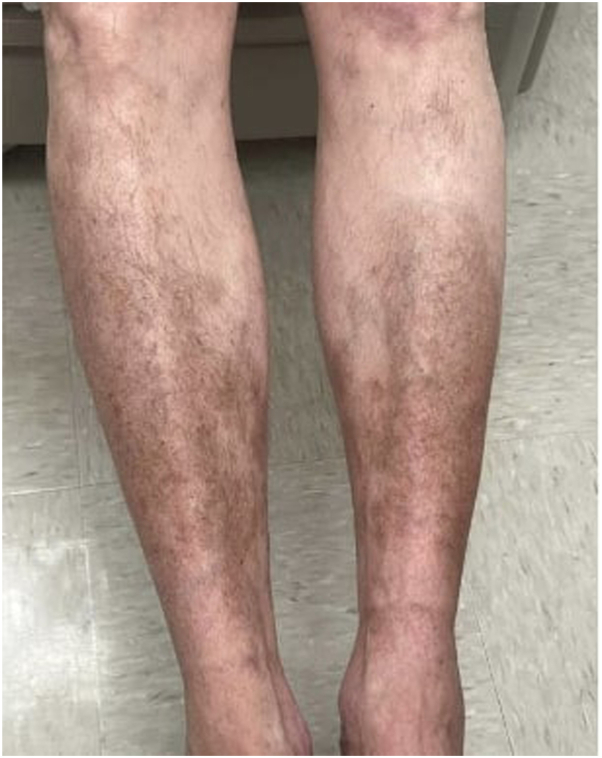
Clinical image of Patient 2, demonstrating speckled lentigines within hyperpigmented patches at the site of previous psoriatic plaques on the bilateral shins.

A 62-year-old man with FST II and a history of ankylosing spondylitis presented with a 5-year history of severe psoriasis. The patient also had a history of squamous cell carcinoma in situ of the tongue with subsequent vocal cord paralysis status post radiation therapy, complicated by recurrent aspiration pneumonia requiring intubation, now with a gastrostomy tube. Prior treatments, including topicals and NB-UVB phototherapy, yielded minimal improvement. He was initially resistant to initiating systemic therapy due to recent admissions for aspiration pneumonia. The patient had widespread psoriatic plaques affecting his scalp, arms, trunk, and lower legs with body surface area (BSA) 12%. After discussion of risks versus benefits, the patient was initiated on risankizumab (150 mg every 12 weeks), achieving near-complete clearance (BSA 1%) within 6 months. On PE, hyperpigmented patches with multiple scattered brown macules in areas of previous psoriasis plaques were observed on the bilateral shins (Fig 2).

### Case 3

**Fig 3:**
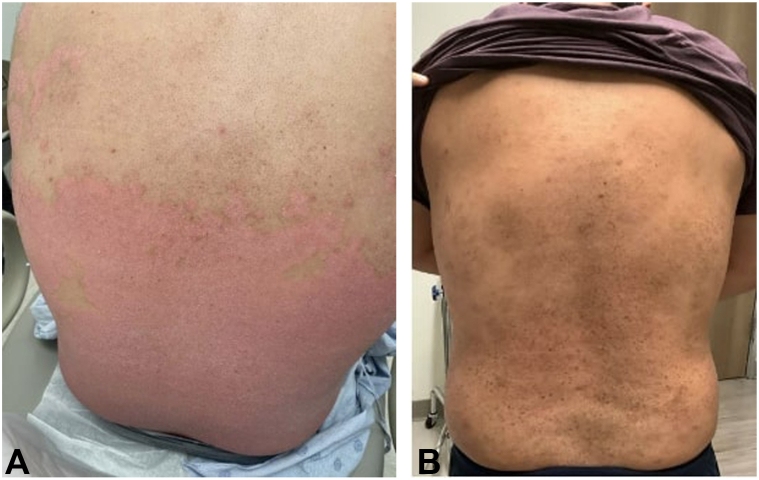
Clinical images of Patient 3, demonstrating psoriatic plaques on the back prior to initiation of ixekizumab therapy **(A)**. One month later, speckled lentigines at the site of previous psoriatic plaques were observed on the back **(B)**.

A 41-year-old man with FST III and a history of anxiety, obesity, and PsA presented with a 15-year history of severe recalcitrant psoriasis. His disease initially involved over 60% BSA, including the scalp, arms, trunk, and legs (Fig 3, *A*). Prior treatments with topicals and home NB-UVB phototherapy provided partial relief. The patient was trialed on guselkumab, but did not meet treatment goal. Four months later, ixekizumab was initiated (80 mg every 4 weeks), leading to significant improvement within 1 month. On PE, the skin was nearly clear (BSA <1%). Multiple hyperpigmented patches with scattered 2-4 mm tan to dark brown macules were observed in areas previously affected by psoriatic plaques, particularly on the forearms, legs, and back (Fig 3, *B*).

### Case 4

A 37-year-old man with FST III and a history of HLD, nonalcoholic fatty liver disease, and obesity presented with a 10-year history of severe psoriasis (BSA 25%) affecting the face, scalp, arms, trunk, and legs. His disease had been refractory to topicals, NB-UVB phototherapy, and apremilast, the latter of which was discontinued due to gastrointestinal intolerance. The patient was initially hesitant to start injection medications due to fear of needles and adverse effects but was eventually initiated on several biologic therapies, guselkumab, ixekizumab, and risankizumab, for months at a time with limited success. Ultimately, he was switched to bimekizumab (160 mg every 4 weeks), achieving complete clearance within 3 months (BSA 0%). On PE, multiple dark brown macules within patches of hyperpigmentation were noted on the right arm and abdomen, corresponding to areas of previous psoriatic plaques.

### Case 5

**Fig 4:**
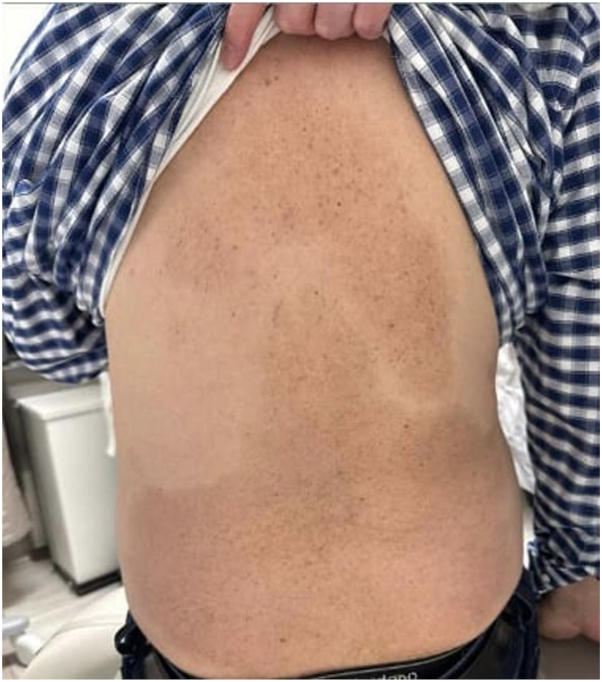
Clinical images of patient 5, demonstrating speckled lentigines within hyperpigmented patches at the site of previous psoriatic plaques on the back.

A 70-year-old man with FST III and a history of cerebrovascular accident, HLD, HTN, NAFLD, obesity, a 30-year history of severe psoriasis (BSA 30% to 40%) of the arms, trunk, and legs, and severe PsA requiring use of a cane, presented for follow-up. His disease had been refractory to topicals, NB-UVB phototherapy, and apremilast. Over the course of several years, the patient was treated with multiple biologic agents. Adalimumab resulted in improvement of his joint symptoms but had minimal effect on cutaneous disease, secukinumab provided no appreciable cutaneous benefit, and ixekizumab was discontinued due to worsening PsA. The patient was subsequently initiated on guselkumab (100 mg every 8 weeks), with deucravacitinib (6 mg daily) added 3 months later. Near-complete skin clearance (BSA <1%) was achieved after 1 year on guselkumab, following the addition of deucravacitinib. On PE, multiple light to dark-brown macules within patches of hyperpigmentation were noted on the trunk, corresponding to areas of previous psoriatic plaques (Fig 4). Notably, the presence of lentigines was documented before the addition of deucravacitinib.

### Case 6

**Fig 5:**
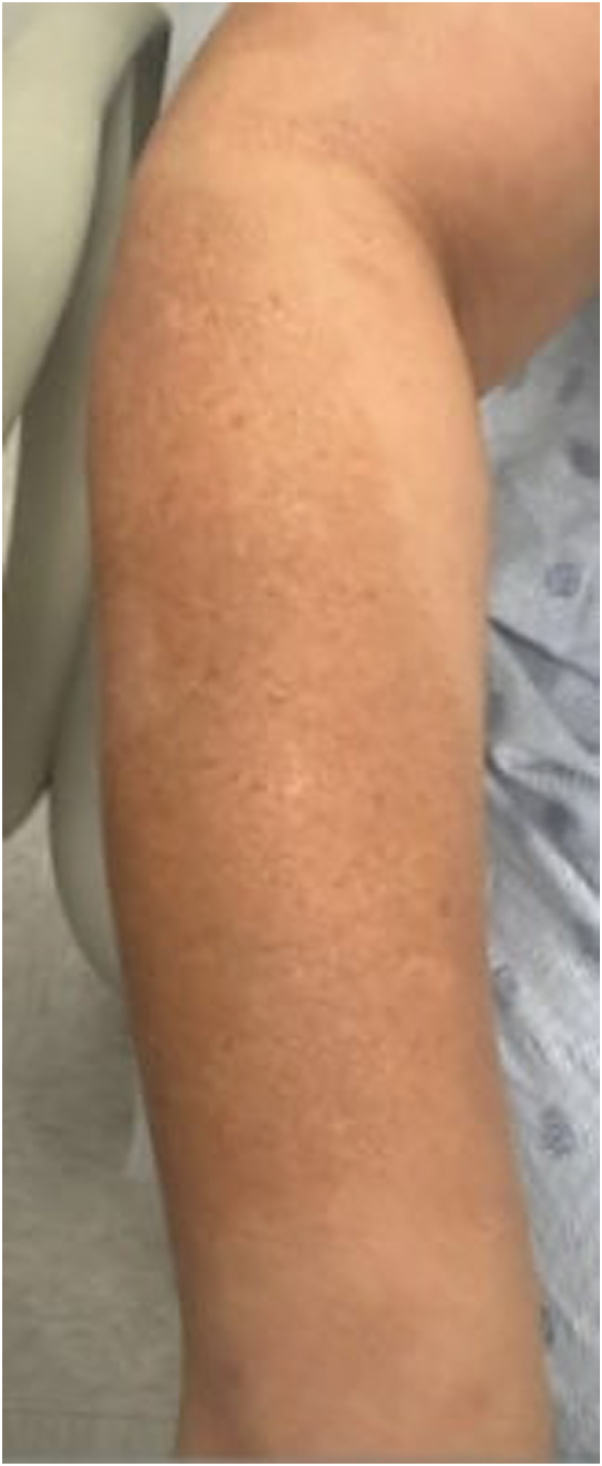
Clinical images of patient 6, demonstrating multiple lentigines within hyperpigmented patches at the site of previous psoriatic plaques on the right forearm.

A 44-year-old female with FST IV and a history of anxiety, HLD, and PsA presented with a 20-year history of severe psoriasis with involvement of her scalp, posterior ears, extremities, and trunk. Prior treatments included topicals, NB-UVB phototherapy, etanercept, adalimumab, secukinumab, and ixekizumab, which provided minimal improvement. Over the past year, the patient was on low-dose methotrexate and guselkumab (100 mg every 8 weeks), achieving significant disease control with a reduction in BSA involvement from greater than 20% to 1%. On PE, multiple lentigines within hyperpigmented patches were noted on the bilateral arms in areas of previous psoriatic plaques (Fig 5).

## Discussion

In this case series, we present 6 patients who developed ELRP following treatment with IL-17A or IL-23 inhibitor biologics (Table I). All patients had a history of severe psoriasis and were noted to have lentigines within 6 months of initiating biologic therapy. To the best of our knowledge, this represents the first documented case of ELRP associated with bimekizumab and the second case reported following ixekizumab treatment. With the increasing use of biologics for psoriasis, our case series and updated review expand the spectrum of biologics associated with ELRP.

**Table I:** Characteristics of patients with eruptive lentiginosis in resolving psoriatic plaques following biologic therapy

Case	Biologic, therapeutic target (dosage)	Age, Sex	FST	PsO hx (y)	Sites of PsO involvement	Location of lentigines	Comorbidities∗	Prior therapies tried	Hx of phototherapy†	Initial BSA (%)	BSA on current therapy (%)	Timing of ELRP onset (mo)
1	Secukinumab, IL-17A (150 mg every 4 wk)	51, F	III	10	Scalp, neck, arms, trunk, legs	Forearms, elbows	Anx, HLD, HTN, Obes, PsA	Topicals, acitretin, adalimumab	Yes	Unk	0	<12
2	Risankizumab, IL-23 (150 mg every 12 wk)	62, M	II	5	Scalp, trunk, legs	Shins	None	Topicals	Yes	12	1	<12
3	Ixekizumab, IL-17A (80 mg every 4 wk)	41, M	III	15	Scalp, arms, trunk, legs	Arms, trunk, legs	Anx, Obes, PsA	Topicals, guselkumab	Yes	>60	<1	1
4	Bimekizumab, IL-17A and IL-17F (160 mg every 4 wk)	37, M	III	10	Face, scalp, arms, trunk, legs	Right arm, abdomen	HLD, NAFLD, Obes	Topicals, apremilast, guselkumab, ixekizumab, risankizumab, bimekizumab	Yes	25	0	1.5
5	Guselkumab, IL-23 (100 mg every 8 wk)	70, M	III	30	Arms, trunk, legs	Trunk	CVA, HLD, HTN, NAFLD, Obes, PsA	Topicals, apremilast, adalimumab, secukinumab, ixekizumab	Yes	30-40	<1	2
6	Guselkumab, IL-23 (100 mg every 8 wk)	44, F	IV	20	Scalp, posterior ears, arms, trunk, legs	Arms, trunk, legs	Anx, HLD, PsA	Topicals, etanercept, methotrexate, adalimumab, secukinumab, ixekizumab	Yes	>20	1	2

*Anx*, Anxiety; *BSA*, body surface area; *CVA*, cerebrovascular accident; *ELRP*, eEruptive lentiginosis arising in areas of resolving psoriatic plaques; *FST*, Fitzpatrick skin type; *HLD*, hyperlipidemia; *HTN*, hypertension; *Hx*, history; *IL*, interleukin; *NAFLD*, nonalcoholic fatty liver disease; *Obes*, obesity; *PsA*, psoriatic arthritis; *PsO*, psoriasis; Unk, unknown.

∗ Comorbidities include: abdominal aortic aneurysm, anxiety, aortic valve stenosis, atrial fibrillation, cerebrovascular accident, coronary artery disease, depression, hyperlipidemia, hypertension, inflammatory bowel disease, myocardial infarction, nonalcoholic fatty liver disease, obesity, psoriatic arthritis, rheumatoid arthritis, Sjogren's syndrome, systemic lupus erythematosus, systemic sclerosis, type 2 diabetes mellitus, and uveitis.

† All patients with a history of phototherapy received narrowband ultraviolet B (NB-UVB). No other phototherapy modalities were reported.

A comprehensive literature review was conducted using PubMed, Google Scholar, and reference tracking from identified articles. This search, including our 6 cases, yielded a total of 33 cases (Table II). The average patient age was 40.67 years (range: 9-75 years), with a male predominance (20/33, 60.6%). The average history of psoriasis was 16.18 years, excluding 11 patients (11/33, 33.3%) with missing data. FST was documented in 19 cases (19/33, 57.6%) and unspecified in 14 cases (14/33, 42.4%). Of the documented 19 cases, skin type III was the most common (12/19, 63.2%), followed by type II (3/19, 15.8%), and type IV (3/19, 15.8%). One case had skin type III-IV (1/19, 5.26%).

**Table II:** Summary of all reports describing eruptive lentiginosis in resolving psoriatic plaques following biologic therapy

Biologic, therapeutic target (dosage)	Reference	Age, Sex	FST	PsO hx (y)	Sites of PsO involvement	Location of lentigines	Comorbidities∗	Prior therapies tried	Hx of photo-therapy (type)	Histo- pathology
Adalimumab,TNF-α (40 mg every 15 d)	Santos-Juanes et al^10^	55, F	Unk	25	Elbows, knees	Elbows, knees	PsA	Topicals, acitretin, adalimumab	No	Consistent with lentigines
Adalimumab, TNF-α (Unk)	Garcia-Souto^11^	45, F	Unk	Adolescence	Back	Back	None	Topicals, methotrexate, cyclosporine	No	Consistent with lentigines
Adalimumab,TNF-α (40 mg every other wk)	Yamamoto^6^	55, M	Unk	6	Unk	Lower extremities	PsA	Topicals	No	Unk
AdalimumabTNF-α (Unk)	Di Cesare^12^	3 of 6 pts (2 F, 4 M; Median age 64; Range: 50-75)	Unk	Unk	Unk	In sites of previous psoriasis plaques	5 of 6 pts with PsA	Topicals, systemic treatments (5/6 cyclosporine, 3/6 methotrexate, 1/6 retinoids)	Unk	Biopsy in 1 of 3 pts: Consistent with lentigines
Bimekizumab, IL-17A and IL-17F (160 mg every 4 wk)	Current study	37, M	III	10	Face, scalp, arms, trunk, legs	Right arm, abdomen	HLD, NAFLD, Obes	Topicals, apremilast, guselkumab, ixekizumab, risankizumab, bimekizumab	Yes (NB-UVB)	Not performed
Etanercept,TNF-α (50 mg twice weekly for 12 wk, then 25 mg twice weekly)	Costa et al^13^	30, M	III	15	Unk	Abdominal area	Unk	Topicals, methotrexate, cyclosporine, acitretin	Yes (PUVA)	Consistent with lentigines
Etanercept,TNF-α (50 mg twice weekly for 12 wk, then 25 mg twice weekly)	Costa et al^13^	50, F	III	25	Unk	Abdominal area, thighs	Unk	Topicals	Yes (UVB)	Unk
Etanercept, TNF-α (50 mg twice weekly)	Almazán-Fernández^14^	60, M	Unk	40	Unk	In sites of previous psoriasis plaques	HTN	Topical and systemic therapies	No	Consistent with lentigines
Etanercept, TNF-α (50 mg twice weekly)	Almazán-Fernández^14^	53, F	Unk	30	Unk	Forearms, buttocks, legs	Unk	Topicals, methotrexate, acitretin	No	Consistent with lentigines
Etanercept, TNF-α (50 mg weekly)	LaRosa et al^15^	9, M	III	5	Scalp, groin, scattered throughout body	Posterior auricular regions, trunk	Unk	Topicals, acitretin, methotrexate	Yes (NB-UVB)	Unk
Etanercept, TNF-α (50 mg twice weekly)	LaRosa et al^15^	12, F	II	6	Arms, trunk, legs, feet	Elbows, knees	Unk	Topicals	Yes (NB-UVB)	Unk
Etanercept,TNF-α (50 mg twice weekly)	LaRosa et al^15^	16, M	III	9	Non-sun exposed area throughout body	Chest and lower back	Unk	Topicals	No	Unk
Etanercept,TNF-α (Unk)	Di Cesare^12^	1 of 6 pts (2 F, 4 M; Median age 64; Range: 50-75)	Unk	Unk	Unk	In sites of previous psoriasis plaques	5 of 6 pts with PsA	Topicals, systemic treatments (5/6 cyclosporine, 3/6 methotrexate, 1/6 retinoids)	Unk	Unk
Guselkumab, IL-23 (100 mg every 8 wk)	Lee et al^16^	41, M	IV	15	Scalp, arms, legs	Arms, legs	Unk	Topicals	Unk	Unk
Guselkumab, IL-23 (Unk)	Kilgore et al^2^	36, F	Unk	Unk	Arms, trunk, legs	Arms, trunk, legs	Unk	Methotrexate, adalimumab	Unk	Not performed
Guselkumab, IL-23 (100 mg every 8 wk)	Current study	70, M	III	30	Arms, trunk, legs	Trunk	CVA, HLD, HTN, NAFLD, Obes, PsA	Topicals, apremilast, adalimumab, secukinumab, ixekizumab	Yes (NB-UVB)	Not performed
Guselkumab, IL-23 (100 mg every 8 wk)	Current study	44, F	IV	20	Scalp, posterior ears, extremities, trunk	Arms, trunk, legs	Anx, HLD, PsA	Topicals, etanercept, methotrexate, adalimumab, secukinumab, ixekizumab	Yes (NB-UVB)	Unk
Infliximab, TNF-α (5 mg/kg IV for 8 wk after 0 and 2 wk infusions)	Dogan and Atakan^17^	55, F	III	12	Scalp, trunk, arms, legs	Back, left arm, legs	Unk	Topicals, methotrexate, adalimumab	Unk	Unk
Infliximab, TNF-α (Unk)	Di Cesare^12^	1 of 6 pts (2 F, 4 M; Median age 64; Range: 50-75)	Unk	Unk	Unk	In sites of previous psoriasis plaques	5 of 6 pts with PsA	Topicals, systemic treatments (5/6 cyclosporine, 3/6 methotrexate, 1/6 retinoids)	Unk	Unk
Ixekizumab,IL-17A (160 mg every 4 wk)	Maria et al^18^	56, M	III	10	Unk	Arms, trunk	T2DM, HLD, HTN	Topicals, methotrexate	Unk	Consistent with both postinflammatory hyperpigmentation and lentigines
Ixekizumab, IL-17A (80 mg every 4 wk)	Current study	41, M	III	15	Scalp, arms, trunk, legs	Arms, trunk, legs	Anx, Obes, PsA	Topicals, guselkumab	Yes (NB-UVB)	Not performed
Risankizumab, IL-23 (150 mg every 12 wk)	Palmisano et al^4^	30, F	Unk	10	Back, arms, legs	Arms	Unk	Cyclosporine, adalimumab	Unk	Reflectance confocal microscopy was consistent with lentigines
Risankizumab, IL-23 (150 mg every 12 wks)	Current study	62, M	II	5	Scalp, trunk, legs	Shins	None	Topicals	Yes (NB-UVB)	Not performed
Secukinumab, IL-17A (Unk)	Uzancakmak et al^7^	37, M	III	2	Trunk, arms, legs	Unk	None	Unk	No	Consistent with lentigines
Secukinumab, IL-17A (Unk)	Uzancakmak et al^7^	38, F	III	4	Unk	Elbows, knees	None	Etanercept, adalimumab	No	Consistent with lentigines
Secukinumab, IL-17A (150 mg every 4 wk)	Current study	51, F	III	10	Scalp, neck, arms, trunk, legs	Forearms, elbows	Anx, HLD, HTN, Obes, PsA	Topicals, acitretin, adalimumab	Yes (NB-UVB)	Not performed
Tildrakizumab, IL-23 (100 mg every 12 wk)	Khanna et al^5^	50, M	Unk	4	Unk	Anterior trunk, left leg	PsA, T2DM	Topicals, methotrexate, adalimumab	Unk	Unk
Ustekinumab, IL-12 and IL-23 (45 mg every 12 wk)	Gutierrez- Gonzalez^19^	40, M	IV	15	Unk	Trunk, extremities	Unk	Topicals, methotrexate, acitretin	No	Not performed
Ustekinumab, IL-12 and IL-23 (150 mg every 4 wk)	Micieli et al^3^	29, M	III-IV	6	Scalp, trunk, arms	Trunk, arms	None	None	No	Consistent with lentigines
Ustekinumab, IL-12 and IL-23 (Unk)	Parietti et al^8^	48, M	II	Adolescence	Trunk, arms, legs	Back, abdomen, thighs	None	Topicals, methotrexate	No	Not performed
Ustekinumab, IL-12 and IL-23 (Unk)	Di Cesare^12^	1 of 6 pts (2 F, 4 M; Median age 64; Range: 50-75)	Unk	Unk	Unk	In sites of previous psoriasis plaques	5 of 6 pts with PsA	Topicals, systemic treatments (5/6 cyclosporine, 3/6 methotrexate, 1/6 retinoids)	Unk	Unk

*Anx*, Anxiety; *CVA*, cerebrovascular accident; *FST*, Fitzpatrick skin type; *HLD*, hyperlipidemia; *HTN*, hypertension; *Hx*, history; *IL*, interleukin; *NB-UVB*, narrow band ultraviolet B; *Obes*, obesity; *PsA*, psoriatic arthritis; *PsO*, psoriasis; *Pt*, patient; *PUVA*, psoralen and ultraviolet A; *T2DM*, type 2 diabetes mellitus; *TNF-α*, tumor necrosis factor alpha; *UVB*, ultraviolet B; Unk, unknown.

∗ Comorbidities include: abdominal aortic aneurysm, anxiety, aortic valve stenosis, atrial fibrillation, cerebrovascular accident, coronary artery disease, depression, hyperlipidemia, hypertension, inflammatory bowel disease, myocardial infarction, nonalcoholic fatty liver disease, obesity, psoriatic arthritis, rheumatoid arthritis, Sjogren's syndrome, systemic lupus erythematosus, systemic sclerosis, type 2 diabetes mellitus, and uveitis.^20^, ^21^, ^22^, ^23^, ^24^

A detailed evaluation of comorbid conditions was performed among the 33 patients included in our analysis. Eighteen patients (18/33, 54.5%) were found to have at least 1 comorbidity. The most prevalent was PsA, observed in 11 patients (11/18, 61.1%), followed by HLD in 5 patients (5/18, 27.8%), HTN in 4 patients (4/18, 22.2%), obesity in 4 patients (4/18, 22.2%), and anxiety in 3 patients (3/18, 16.7%). Six patients (6/33, 18.2%) had no reported comorbidities, while 9 patients (9/33, 27.3%) had incomplete documentation (Table II).

Among the biologics implicated, etanercept was the most frequently reported (8/33, 24.2%), followed by adalimumab (6/33, 18.2%), guselkumab (4/33, 12.1%), ustekinumab (4/33, 12.1%) and secukinumab (3/33, 9.1%). Notably, 33.3% of patients (11/33, 33.3%) had previously been treated with at least 1 other biologic before ELRP was noted on their current therapy. A history of phototherapy was documented in 10 patients (10/33, 30.3%), out of which NB-UVB was used for 9 patients (9/33, 27.3%) and PUVA for 1 patient (1/33, 3.0%), while the remaining 33.3% (11/33, 33.3%) had no history of phototherapy and 12 (12/33, 36.4%) cases did not mention phototherapy. All 11 patients (11/33, 33.3%) who had a biopsy showed histopathology consistent with lentiginous proliferation.^3^^,^^4^^,^^7^^,^^10^, ^11^, ^12^, ^13^, ^14^^,^^18^

Several hypotheses have been proposed to explain the development of ELRP. Wang et al described the role of IL-17 and tumor necrosis factor alpha (TNF-α) in the suppression of melanogenesis, suggesting that inhibition of these cytokines through biologics may release this suppression, increasing melanocyte activity and lentigine formation.^25^ Similarly, IL-23 has been implicated in pigment regulation via its influence on Th17 cells and downstream suppression of IL-6, an antimelanogenic cytokine. These mechanisms have also been proposed to explain biologic-induced pigmentary changes, such as hair repigmentation following ustekinumab treatment.^9^^,^^11^^,^^16^

Another explanation involves the role of chronic skin damage and subsequent reparative melanocyte activation. All patients in our series had longstanding, severe psoriasis that was either refractory to prior therapies or inadequately managed. In 3 cases, treatment initiation was delayed because of fears related to infection risk (Patient 2), injection-related anxiety (Patient 4), or concern for PsA exacerbation (Patient 5), resulting in prolonged inflammation. Following initiation of biologic therapy, all patients experienced rapid resolution. This abrupt shift in the inflammatory microenvironment, marked by withdrawal of melanogenesis-inhibitory cytokines such as IL-17 and TNF-α, may lead to localized melanocyte hyperactivity and lentigine development confined to previously affected plaques. Although psoriasis is not traditionally considered a scarring condition, our observations suggest that in severe or prolonged disease, permanent pigmentary alterations may occur, highlighting the importance of timely and effective disease management.^3^^,^^7^

Our comorbidity findings align with known psoriasis epidemiology, with the exception of PsA, which appeared more frequently in our series (11/33, 33.3%) than is typically reported (7% to 26%).^26^ This likely reflects the high severity of disease in our patients, as indicated by high BSA involvement. Although BSA was infrequently reported in the cases identified during our literature review, higher BSA is a well-established risk factor for PsA, suggesting that patients with longstanding, extensive psoriasis may have an elevated risk of PsA.^27^ In both the literature and our cases, lentigines persisted with minimal improvement for several years after onset, in some instances lasting up to 5 years.^3^ Partial clearance has been reported in a case of ELRP treated with Q-switched ruby laser, performed 4 years after initial presentation.^3^

Lastly, it is worth noting that all patients in our series had a history of phototherapy. While ultraviolet induced melanogenesis is well-documented, our patients first developed lentigines after rapid clearance of psoriasis with biologic therapy, rather than immediately after phototherapy. Lentigines induced by phototherapy (especially psoralen plus ultraviolet A) are more widespread and not restricted to the areas of prior psoriatic plaques.^14^ This raises the question of whether prior UV exposure primes melanocytes for enhanced activation in the context of immune modulation induced by biologics.

That being said, one-third of patients in our literature search did not receive phototherapy, suggesting phototherapy is a contributory rather than a required factor for ELRP development. Interestingly, all cases were confined to medium skin types (FST II-IV), reflecting potential differences in melanocyte responsiveness. Further research is needed to elucidate potential contributing factors and determine whether ELRP confers an increased risk of actinic damage or malignancy.

In summary, our case series expands the clinical spectrum of ELRP and reinforces its association with a broad range of biologic agents, including IL-17A and IL-23 inhibitors. Key risk factors may include longstanding, severe plaque psoriasis, high BSA involvement, prior UV exposure, and FST II-IV. These findings suggest ELRP is a pigmentary response unique to the resolution of chronic inflammatory skin disease under biologic treatment, most likely driven by sudden cytokine shifts and localized melanocyte hyperactivation. Recognizing this phenomenon is essential for dermatologists managing patients with moderate-to-severe psoriasis, especially as the therapeutic landscape continues to evolve.

## Conflicts of interest

Dr Min is an advisor or consultant for BMS, Priovant; she is an investigator for Amgen, AZ, BMS, BI, and Priovant. Drs Phong, Yale, Golbari, Zachary, Choe, and Author Patel have no conflicts of interest to declare.
